# Pipettes and Pipelines: The Weapons of Omics Sciences for a New Age of Clinical Studies

**DOI:** 10.3390/cimb48070734

**Published:** 2026-07-18

**Authors:** Ícaro S. Lopes, Eduardo R. Fukutani, Tiago F. Mota, Bruno B. Andrade, Mariana Araújo-Pereira, Artur T. L. Queiroz

**Affiliations:** 1 Laboratório de Pesquisa Clínica e Translacional, Instituto Gonçalo Moniz, Fundação Oswaldo Cruz, Salvador 40296-710, Brazil; 2 The SYBIL Lab, Multinational Organization Network Sponsoring Translational and Epidemiological Research (MONSTER) Initiative, Salvador 41720-200, Brazil; 3 Programa de Pós-Graduação em Biotecnologia e Medicina Investigativa, Instituto Gonçalo Moniz, Fundação Oswaldo Cruz, Salvador 40296-710, Brazil; 4 The Genios Lab, Multinational Organization Network Sponsoring Translational and Epidemiological Research (MONSTER) Initiative, Salvador 41720-200, Brazil; 5 The BRIGHT Lab, Multinational Organization Network Sponsoring Translational and Epidemiological Research (MONSTER) Initiative, Salvador 41720-200, Brazil

**Keywords:** omics, clinical studies, review

## Abstract

The omics sciences represent a revolution for clinical studies, offering integrative approaches to analyzing biological data with unprecedented depth. From the discovery of the double-helix structure of DNA to the CRISPR-Cas9 gene editing tool, passing through the evolution of sequencing platforms and the exponential advance of computing power and in silico tools, omics has progressed in its role of leading innovative solutions for old challenges in health sciences. In this review, we describe different omics, the history of their techniques and technologies, data analysis and up-to-date visualization tools used for clinical data in research and health systems. We also discuss how omics are currently being applied in diagnosis, precision and personalized medicine. For the future, omics vow to underpin the majority of decisions made by health professionals, allowing individualized treatments based on Big Data and personal biological information.

## 1. Introduction

The development of new technologies and the evolution of scientific knowledge are mutually reinforcing processes. Over the centuries, clinical practices have been improved based on technological innovations. From time to time, a major technological breakthrough is made, allowing scientists to obtain previously inaccessible data and providing health professionals with new and vast information to prevent and treat many health conditions. The invention of the microscope in the 17th century enabled the emergence of microbiology and led to substantial progress in the knowledge of living beings, permanently altering clinical practices ever since [1]. Less than two centuries ago, the cultivation of microorganisms in the laboratory took biology even further with the possibility of in vitro experiments [2]. Another milestone occurred in the middle of the 20th century, when the double-helix structure of DNA was discovered, paving the way for an unprecedented advance in knowledge of the biological mechanisms that sustain life and rule interactions between beings.

However, it was only about 20 years later that methods capable of deciphering the exact sequence of nucleotides within DNA molecules began to emerge, marking a profound transformation in biomedical sciences [3,4]. This advance provided molecular biologists with accurate and increasingly robust information on the genetic composition of organisms, culminating in the completion of genomes of organisms of different sizes and complexities [5,6]. From this accumulation of data of a new nature, genomics emerged, which would serve as a paradigm for the emergence of a new field of science: the omics sciences.

The term omics is derived from the Greek suffix “-ome”, which gives a sense of collective or completeness, although some authors also associate the term with the Sanskrit “om”, meaning fullness [7,8]. This term summarizes the central concept: the study of molecules, structures, biological and physiological processes from a systemic and integrative approach [9]. The broad view of organisms and systems provided by the omics sciences offers greater sensitivity and efficiency compared to previously used methods, as well as enabling insights and solutions that would not have been possible with any other old technique [7]. Several issues addressed by clinical studies would greatly benefit from the new possibilities offered by omics, generating solutions that would accelerate health sciences in the coming decades.

The discovery of new drugs and the prospecting of new substances with pharmacological potential was a significant challenge in the pre-omics era. Carrying out laboratory experiments as the sole source of this knowledge was costly in time and money and sometimes insufficient in quality, limiting scientific productivity in the field of pharmacology [10]. Plants, whose metabolites are among the main substances with therapeutic properties, have long been organisms with underutilized pharmacological potential due to these operational difficulties [11]. In addition to prospecting for these molecules or compounds, tests to assess the safety and toxicity of possible drugs were also less efficient, agile and accurate using only traditional methods [12].

Similarly, diagnostic methods prior to the emergence of omics left something to be desired in terms of accuracy, speed, work rate and optimization of the use of laboratory resources. Clinical evaluation by the health professional based on the individual’s report and outpatient tests carried greater weight and fed vaguer and more inaccurate diagnoses. Diseases caused by bacterial, fungal and viral pathogens had classic diagnostic methods with several limitations that impacted the individual’s chance of recovery. Cultivation-dependent methods, such as in vitro culture for bacteria and fungi, agar gel immunodiffusion and egg or tissue culture inoculation for viruses, required several days to perform, large laboratory manpower and expensive reagents. ELISA, used to diagnose various types of disease, requires specific antibodies for each type of pathogen or molecular marker, setting the precedent for misidentification and increasing the cost of performing it [13].

## 2. Lord of the Omics: The Two Machines—High-Throughput Multi-Omics Methodologies

### 2.1. Genomics

The advent of DNA sequencing was the propeller of the whole field of genomics, and their progresses have been walking together ever since (Table 1). Currently, the best established and prolific omic science, genomics, consists of the study of the whole genome of an organism and comprises the description, characterization and comparison of genes, single nucleotide and copy number variations, structure and other features [14,15]. From the first complete DNA genome sequenced, the bacteriophage λ, in 1982, to the completion of the Human Genome Project, 21 years later, many technical and technological improvements paved the way to modern genomic analysis and provided scientists with a huge amount of data that changed previous conceptions of human genetic nature [5,6]. Genomic analysis has revealed the high degree of identity between the human genome and that of other beings, the importance of non-coding DNA and the regulation of gene expression in the complexity of organisms and the role of single nucleotide polymorphisms in the occurrence of genetic diseases in humans, for example. It was also possible to understand the genetic mechanisms involved in important pathogenic processes, such as bacterial resistance to antibiotics, adaptation of pathogens to the host’s immune system and the genomic plasticity of intracellular pathogens [16,17,18].

Although DNA sequencing is steadily evolving and is now capable of generating sequences of tens of thousands of nucleotides, assembly is still a necessary step in order to obtain complete genomes of more complex organisms. Through the years, assembly algorithms moved on from the time-consuming, input size-limiting overlap-layout-consensus strategy, which compares all DNA reads against each other in an effort to find overlaps, to the more complex graph approach, based on the division of reads into shorter k-mers to be compared and then ordered along with adjacent k-mers sequences. This latter strategy can better handle the larger datasets generated by modern high-throughput sequencing platforms, while the former is still useful for TGS data [19]. Among the most commonly used genome assembly software for short-read sequencing are SPAdes [20], Velvet [21], ABySS [22] and SOAPdenovo2 [23]. For long-read sequencing, Canu [24], Falcon [25] and HGAP [26] are the most common choices.

After obtaining complete genomes, an essential step in characterizing individuals is variant analysis or variant calling, which comprise the unique sequences that differentiate them from one another. This analysis, which can also be applied directly to read libraries from sequencing, depends on the alignment of the genome of interest or mapping of reads against a reference genome, allowing the identification of variable loci between individuals. This knowledge revealed the role of variants in hereditary and other diseases, such as cancer [27,28]. In clinical studies, three different sequencing strategies can be applied to perform variant analysis: single and multi-gene panels, which target only a group of genes of interest for sequencing; exome sequencing, which targets the entire group of protein-coding genes; and genomic sequencing, which allows the comparison of any region of the genome in search of variants. Each of these strategies offers advantages for different types of studies, depending on the need for coverage, number of genes to be analyzed, budget, and time available, although all strategies have high sensitivity for variant detection [28]. Bioinformatics tools such as GATK HaplotypeCaller [29] and Platypus [30] are efficient in the computational analysis of this data.

A practical tool for characterizing and obtaining relevant functional information from genomes is comparative genomics. With this, one can refine the core genome and the pan genome using a handful of strategies to analyze a set of genomes [31]. A core genome reveals the set of genes shared by the genomes analyzed, while a pan genome contains the whole set of genes present in the input genomes [32]. These analyses rely on a high-quality annotation reference to correctly identify genes in newly assembled genomes. Computational capability and alignment curation still represent challenges for the generation of new pangenomes, motivating researchers to seek new solutions to optimize the process [33].

Despite being a fundamental step in genome analysis, annotation is still a bottleneck in this process. Almost half of all eukaryotic species assemblies in the NCBI Genbank database still have no annotation, making it more difficult to assess functional information from whole phylogenetic groups, even though there is an increasing availability of genomic data [34,35]. Current annotation strategies are based both on transcripts and protein homology information, the so-called extrinsic evidence, and ab initio statistical models from genomic data, called intrinsic evidence. While intrinsic evidence helps to provide information about gene regions that extrinsic evidence cannot cover, it also needs extrinsic evidence to correct errors caused by the statistical models [35,36]. Pipelines like GeneMark-ETP [36] are largely used for eukaryotic gene annotation using a hybrid approach.

Recent advances have led to the construction of the first reference draft of the human pangenome. This achievement has enabled a reduction in errors in the discovery of small variants and an increase in the number of structural variants detectable in human genomic analyses [33]. Small variants have already been identified as playing a central role in the development and progression of a number of diseases, such as cancer and schizophrenia [37,38]. In turn, advances in knowledge of structural genomic variants have allowed for an increasingly deeper understanding of the mechanisms involved in the emergence of various types of cancer [39,40,41].

### 2.2. Metagenomics

Metagenomics is the study of genetic material directly extracted from environmental or clinical samples without the need for isolating and cultivating individual species in a laboratory. The study of microorganisms progressed from Antonie van Leeuwenhoek’s observations using microscopes in the 17th century to the current definition of microbial communities, i.e., a set of microorganisms coexisting in the same space and time, the development of culturing techniques, and the understanding that these methods did not capture the full range of microbial diversity [42]. Such limitations to culture the microbial community for its assessment as a whole have brought light to new approaches using a marker gene such as 16S rRNA for bacteria and 18S rRNA for fungi and other eukaryotes [43].

These marker genes have brought advantages over the culture-dependent method as they are present in almost all bacteria and eukaryotes, making them universal targets for species identification [44,45]. These genes’ function has remained relatively constant over time, allowing them to serve as a reliable molecular chronometer for phylogenetic studies [45]. Both genes could be amplified using universal Polymerase chain reaction (PCR) primers, and sequencing technologies developed by Sanger and colleagues [46] have made it possible to generate full-length 16S/18S rRNA gene sequences at a relatively low cost. The usage of marker genes for microbial classification has enabled the study of microbial communities directly from environmental samples without the need for culturing. However, studies have highlighted its limitations in providing species-level resolution and the need to account for intragenomic variation in 16S rRNA gene copies within a single genome [47].

The advent of NGS technologies has led to the establishment of metagenomics as a distinct field in the late 20th century, with higher sequence depth in both 16S rRNA and shotgun sequencing of environmental samples without culturing. The usage of shotgun sequencing together with NGS technologies, Whole Genome Sequencing (WGS), granted us the possibility to detect more bacterial species than 16S rRNA sequencing, providing a more comprehensive understanding of microbial diversity [48]. The WGS can also provide more accurate taxonomic resolution at the species level, comprehensive data on microbial functional genes, and capture the entire microbial community, including viruses and fungi, whereas marker gene sequencing is restricted to its organism [49]. The marker gene approach is still used with NGS technologies, which have a lower cost in comparison with WGS and have more established analysis pipelines [50].

The strategy to be employed in a metagenomic study depends on the specific goals of the microbiome study. In order to study the bacterial composition of an environmental sample, 16S sequencing is enough. However, if the question of the study involves the whole microbial structure, i.e., fungi, bacteria, protozoans, and viruses, as well as determining the antibiotic resistance profile and functional characterization of the microbial community, the WGS approach is needed.

Few areas of clinical studies have benefited as much from omics as the study of microbiomes [51]. Global metagenomic sampling projects have helped discover thousands of new microbial species in host and environmental samples [48,52]. Only the broad approaches offered by the integration of “meta-omics” make it possible to infer functional and structural characteristics of microbial communities, from their genetic content to the metabolic networks that are built between microorganisms and the host [53]. From this, it is possible to diagnose, understand and treat various diseases that were previously not understood as systemic conditions and dependent on the composition of microbiomes. The emergence and progression of diseases such as Alzheimer’s, arterial and pulmonary hypertension, intestinal inflammation, and cancer have been described as influenced by the human gut microbiota [54,55,56,57]. Based on this, the concept of “precision nutrition” was coined, designating a nutritional strategy complementary to clinical treatment with the aim of nourishing the individual and creating a healthy environment for healthy microbiota [58].

### 2.3. Epigenomics: Beyond Sequencing

Epigenomics, or the study of epigenetic modifications, chromatin structure, and its influence on gene expression, has revolutionized our understanding of gene regulation and its role in health and disease. Instead of studying modifications to the DNA sequence, epigenomics addresses reversible, heritable changes in gene expression that do not modify the underlying DNA sequence. Such changes are the result of chemical modifications on many layers, including the DNA structure itself, with mechanisms like methylation, chromatin remodeling, proteins that involve the DNA, such as histones, and mechanisms related to non-coding RNAs [59]. Furthermore, epigenetic modifications are quite dynamic to allow organisms to adapt in response to environmental factors, developmental stages and physiological processes [60].

This field began in the 1950s, even before the DNA’s double helix structure was discovered, when a developmental biologist named Conrad Waddington first coined the term “epigenetics”. His first model referred to a landscape in which the gene expression would interact with the environment to determine cell fate during development [61]. Soon after Waddington proposed the epigenetic landscape, one of the first epigenetic mechanisms, DNA methylation, was discovered by Rollin Hotchkiss when he found methylated cytosine in bacterial DNA [62]. By the 1970s, DNA methylation was observed in eukaryotes and associated with gene repression, which suggested that this mechanism plays a critical role in gene expression regulation [63].

Afterwards, several mechanisms of gene expression regulation have been discovered, including DNA hydroxymethylation and carboxylation, as well as histones’ post-translational modifications and their involvement in chromatin conformation. Another identified epigenetic mechanism is the non-coding RNAs, including their functions as gene expression regulators and their post-translational modifications, such as RNA methylation [64].

As for technologies that can measure epigenetic mechanisms in studies nowadays, there are several available for each explored mechanism. The first and most studied mechanism is DNA methylation, as this tech can approach DNA methylation locally or globally. The basis for these technologies relies on DNA bisulfite treatment, as treating genomic DNA with sodium bisulfite results in deamination of unmethylated cytosines to uracil and leaves methylated cytosines intact. This is ideal to quantify methylation with 5 mC, the most frequent type of DNA methylation [65].

Assay for Transposase Accessible Chromatin using sequencing (ATAC-seq) is currently one of the most employed methods for epigenomic analysis and a great improvement from older strategies, since it consists of only two steps and can analyze thousands of cells at a time. It is based on direct in vitro transposition of sequencing adapters into chromatin to assess the availability of genomic locations on open chromatin regions, chromatin compaction at the nucleotide level and interaction of DNA-binding proteins, yielding insights into gene expression regulation under a given condition [66]. The Tn5 transposase, a hyperactive prokaryotic enzyme involved in transposition processes, promotes the insertion of DNA sequencing adapters into the accessible chromatin loci, resulting in both DNA fragmentation of these regions and the tagging of the genome with those adapters. Then, PCR amplification of those fragmented sites generates DNA libraries of open chromatin ready to be sequenced [66].

Advances in epigenomic analysis techniques have enabled us to understand the mechanisms involved in various diseases that were not yet sufficiently understood. Epigenomic approaches have revealed the role of epigenetic alterations in deleterious gene regulation in the onset of autoimmune diseases and cancer [67,68]. One example is the study on the epigenome of CD4+ T cells from patients with systemic lupus erythematosus. Changes in gene regulation caused by epigenetic modifications in loci associated with the disease were discovered, resulting in significant differences in gene expression patterns between affected and healthy individuals. Another contribution came from the association between ATAC-seq and RNA-seq, which enabled the identification of enhancers involved in the onset of systemic lupus erythematosus [69].

### 2.4. Pharmacogenomics

Consisting of the use of DNA, RNA and amino acid sequence data applied to drug development and testing, pharmacogenomics derives and benefits greatly from both genomics and pharmacogenetics [70]. Although the human genome varies by only about 0.4% between two individuals, this variation is sufficient to significantly alter the pharmacological response [71]. Factors associated with pharmacological treatment, such as dosage and substance appropriate for greater effectiveness, susceptibility, and adverse reactions, derive from the genomic characteristics of the individual, whose understanding is made possible by the analysis of genetic variations found [12]. Several studies reinforce the role of these variations in diseases such as diabetes, cancer, psychiatric and cardiovascular diseases [70].

In addition to its intrinsic relationship with genomics, through which genetic variants can be identified, pharmacogenomics also uses data derived from other omics to investigate all aspects associated with individualized pharmacological response. The use of RNA-seq to evaluate gene expression patterns, protein structural analysis techniques for observing isoforms, and methods for identifying methylated sites in the genome to obtain epigenomic regulation patterns are also essential for a deep and systemic view of the possible effects of drugs on individuals, allowing for much more accurate prediction of risk and therapeutic response [72].

Advances in pharmacogenomics have opened up new possibilities for innovative treatments for a range of clinical conditions, with the application of stem cells, gene editing, engineered tissues, molecular therapy with DNA and RNA associated with other more traditional interventions, following the best strategy according to the individual’s genetic profile [70]. One of the major contributions of this area, which has served as a crucial tool for the development of these strategies, is the identification of biomarkers (Table 2). These molecules allow us to predict the occurrence and effectiveness of treatment for different conditions, such as genetic and cardiovascular diseases and cancers [73,74]. This approach paves the way for the implementation of personalized medicine and precision medicine, which have attracted great interest from researchers and health professionals in recent years.

### 2.5. RNA Sequencing, Transcriptomics and Metatranscriptomics

The study of the structure and sequence of DNA was a major turning point in the field of molecular biology, earning humanity techniques and knowledge to understand the very backbone of life. Following the processes of molecular biology, the transcription of DNA into RNA and its translation into proteins is how cells can modulate their responses to the environment and exercise their functions [75]. The transcriptome is the study of all RNA transcripts and their quantities, including coding and non-coding RNAs; it is a way of understanding how the genome’s expression is being modulated and how it affects cells, tissues, and even whole organisms’ functions. Since the RNA molecule is unstable and susceptible to degradation, the use of reverse transcriptase was a crucial breakthrough to enable the study of the transcriptome [76].

The study of transcriptomics started in the 90′s, more precisely 1995, with the introduction of the Serial Analysis of Gene Expression (SAGE). This technology is based on Sanger sequencing short identifying sequence tags, usually of 9 to 10 bp, cleaved from defined positions within the transcripts’ cDNA [77,78]. As the probes employed in SAGE did not require previously knowing the measured genes, it was used mainly in the discovery of new genes and studies approaching functional genomics [79]. However, this technology was overthrown by microarray, which had its debut in the early 2000s and is one of the most prominent technologies in gene expression studies to date. Instead of relying on short sequence tags, microarrays rely on hybridization between known target genes’ cDNA marked with fluorescence and probes fixed on their templates, measuring tens of thousands of genes by the light emitted at hybridization [80]. Despite the necessity of knowing which genes will be measured in microarrays, it is still preferred over SAGE due to its superior practicality, cost-effectiveness, extensive tools for analyzing data, and gene coverage, which includes known genes, splice variants, and non-coding RNAs [81,82].

In the late 2000s and the beginning of the 2010s, RNA sequencing (RNA-seq) technology with high-throughput platforms was introduced in the market and became the most used method in transcriptomic research [76]. Despite its higher cost compared to microarray, RNA-seq presents a broader dynamic range and higher sensitivity, detecting more transcripts, including the ones with low expression values that would not be detected in microarray [83]. Also, this technology enables the detection of novel transcripts and isoforms, has reduced technical biases, and is also able to detect sequence variations, such as SNPs and gene fusions [84].

In the clinical context, both microarray and RNA-seq are suitable for identifying differentially expressed genes among different conditions (treatment/disease vs control) and also providing additional insights into such conditions. For example, several transcriptomic biomarkers based on the host’s blood gene expression for tuberculosis were previously proposed, exploring both microarray and RNA-seq data [85,86,87]. However, RNA-seq has one more utility than microarray, as it can identify novel transcripts, splice variants, and gene fusions as biomarker candidates [88,89].

The general workflow for transcriptomic data analysis includes data from both the most prominent employed techniques, microarray and NGS, and includes the steps of data preprocessing, normalization, downstream analyses and visualization. For microarray, the preprocessing is far simpler, being based on examination of image plots and histograms to check for outlier samples [90]. Furthermore, the preprocess can also include the removal or mitigation of batch effect when two or more arrays are analyzed together [91,92]. Following, the preprocessed data should be normalized to minimize systematic biases and enable significant comparisons in downstream analyses [93]. Several methods can be employed to normalize microarray data, such as global median normalization, Lowess (locally weighted scatterplot smoothing), Quantile normalization, RMA (robust multi-array average) or log transformation [94].

On the other hand, preprocessing RNA-seq data includes trimming adapters and low-quality reads, aligning and mapping the reads to a reference genome and creating a count table with the gene expression values. Trimming can be performed using software such as Trimmomatic [95]; read alignment and mapping can be performed using Salmon [96], STAR [97], or similar programs, alongside the organism’s reference genome, while creating the count table can be done using the tximport package in R [98]. The normalization of NGS data can be achieved through several methods, including DESeq2′s variance stabilizing transformation [99], quantile normalization and counts per million scaling [100]. Overall data for RNA-seq and microarray can be visualized through boxplots, principal component analysis and heatmaps.

The downstream analysis of transcriptomic data aims to identify differentially expressed genes (DEGs), possible correlations between genes, associated biological pathways, biomarkers capable of discriminating samples by group and even alternative gene splicing. Differential expression analysis is performed by comparing the means of gene expression values between two or more groups (disease/treatment vs control). This can be achieved in microarray through statistical tests, such as *t*-test or ANOVA, or by fitting linear models to the normalized data [101]. RNA-seq data demands methods with models that account for its binomial negative distribution in order to properly identify DEGs, as seen in DESeq2 [101] and edgeR packages [102]. Visualization of differential expression analysis is based on volcano plots, heatmaps and Venn diagrams to display common DEGs among different comparisons.

Next, enrichment analysis can be performed to gain insights into the DEGs’ biological functions, checking for overrepresented functional categories, pathways, or regulatory networks in databases, such as KEGG, Gene Ontology or Reactome. Examples of R packages that can perform enrichment analysis are clusterProfiler [103,104], pathfindR [105] and similar. Enriched pathways can be visualized in barplots, dotplots and even network plots [103,104]. Clustering analysis, such as hierarchical clustering and k-means, can provide additional insights about gene relationships [106,107], while correlation analysis checks for correlations between gene expressions or variables of interest [108], for example, glucose levels in diabetes studies [109]. Clusters are visualized in dendrograms and heatmaps, and correlations can be visualized in network plots, arch diagrams and chord diagrams.

Another focus of downstream analysis is the identification of biomarkers by classification machine learning algorithms, such as support vector machine, randomForest and neural networks [110]. Machine learning’s results are visualized by plotting the variables’ importance values into bar plots or dot plots, while the identified model’s accuracy can be visualized in confusion matrices, ROC curves and heatmaps. Lastly, splicing analysis can be performed to assess which alternative splicing of a gene has been differentially expressed, while also accounting for events such as deletion, insertion, substitution and frame disruption or truncation. LeafCutter and Bisbee are examples of packages that can be employed to perform this analysis [111,112]. Differentially expressed isoforms can be visualized in volcano plots and heatmaps, events are displayed in bar plots or dot plots and reads per region are visualized in sashimi plots [111].

The application of molecular diagnostic methods represented a major improvement over the costly and limited methods of the past. The detection of viral RNAs and proteins in patient samples offers significant gains in sensitivity, specificity, speed, and cost-effectiveness [13]. This type of diagnosis also allows for better integration with epidemiological and treatment strategies, such as identifying variants, predicting antiviral resistance, and monitoring the circulation of the pathogen in large regions, reducing the time needed for authorities and health professionals to take action to control the pathogen. Practical examples of this statement were seen during the H1N1 pandemic in 2009 and the SARS-CoV-2 pandemic in 2020 [13,113,114]. Amid intensive efforts to develop diagnostic kits for COVID-19, a series of molecular methods based on nucleic acid amplification tests (NAAT) were obtained and contributed to the containment of the disease. Of these, the detection of SARS-CoV-2 RNA in respiratory samples using real-time reverse transcriptase—polymerase chain reaction (RTPCR) was successful and came to be considered the gold standard for diagnosis [114,115]. Several other diseases caused by different pathogens are also detected by NAAT, such as tuberculosis, leptospirosis, and a number of sexually transmitted infections [116,117,118,119].

A recent and groundbreaking application of transcriptomics knowledge is the development of vaccines based on nucleic acid molecules, the main successful example of which was the immunization strategy during the SARS-CoV-2 pandemic. Using either DNA or mRNA, these vaccines utilize the host’s own machinery to synthesize antigens that interact with the immune system in the process of promoting an immune response to the pathogen, based on the inoculation of molecules that encode the gene or transcript responsible for these proteins. This strategy has a number of advantages over traditional vaccines: greater safety, as it emulates the inflammatory process without using any part of the pathogen, thus eliminating the risk of causing disease; adaptability, as it allows the DNA or RNA sequence to be modified to update the vaccine according to mutations in the pathogen’s genome; a quick and simple production process, allowing for scaling up to mass production and accelerating the mitigation of epidemics; and high efficacy, due to its ability to induce a strong cellular and humoral response. In addition to viral infections, nucleic acid vaccines are a promising strategy for fighting cancer [120].

### 2.6. Proteomics: Spectrometry and Electrophoresis

Proteomics, the large-scale study of proteins, has emerged as a crucial complement to genomics, providing a more comprehensive understanding of biological systems and disease mechanisms [121]. The origins of proteomics can be traced back to the 1970s, when researchers began exploring techniques for separating and analyzing proteins [122]. One of the earliest breakthroughs was the development of two-dimensional gel electrophoresis (2D-GE), which allowed for the separation of complex protein mixtures based on their isoelectric point and molecular weight [123]. This technique, coupled with the advent of mass spectrometry (MS), paved the way for the identification and quantification of proteins in biological samples [124].

With the proteomics field evolution, the focus shifted from the analysis of individual proteins to the comprehensive study of the entire protein complement of a cell, tissue, or organism, known as the “proteome” [125]. This holistic approach was facilitated by the rapid advancements in MS technology, particularly the introduction of soft ionization techniques such as electrospray ionization (ESI) and matrix-assisted laser desorption/ionization (MALDI) [126,127,128]. These innovations awarded John B. Fenn (ESI) and Koichi Tanaka (soft laser desorption) with the Nobel Prize in Chemistry 2002 and enabled the analysis of intact proteins and peptides, leading to the development of high-throughput proteomic platforms [129].

#### 2.6.1. Mass Spectrometry-Based Proteomics (Targeted and Untargeted)

Proteomic assays can be broadly classified into two main categories: targeted and untargeted approaches [130]. Targeted proteomic assays involve the analysis of a pre-defined set of proteins or peptides of interest. These assays are typically used to study the abundance, modifications, and interactions of specific proteins that are known to be relevant to a particular biological process or disease [131]. Targeted approaches often employ techniques such as selected reaction monitoring (SRM) or multiple reaction monitoring (MRM), which use MS to selectively monitor the transitions of specific precursor–product ion pairs [132].

The evolution of proteomic assays has been driven by significant technological advancements in both analytical instrumentation and computational tools [133]. For instance, mass spectrometry has been at the forefront of these developments, with the introduction of increasingly sensitive and high-resolution instruments [134]. The advent of hybrid mass spectrometers, such as quadrupole-time-of-flight (Q-TOF) and Orbitrap-based instruments, has enabled the acquisition of high-quality MS and tandem MS (MS/MS) data [135], allowing for more accurate protein identification and quantification [136]. Furthermore, the integration of MS with liquid chromatography (LC-MS) has significantly improved the separation and analysis of complex protein and peptide mixtures [137]. To enhance the depth of proteomic coverage, various protein fractionation and enrichment techniques have been developed [138]. These include methods such as protein or peptide chromatography, affinity-based purification, and subcellular fractionation, which help to reduce sample complexity and increase the detection of low-abundance proteins [139].

As proteomic technologies have advanced, the number of molecules that can be evaluated in a single experiment has increased dramatically. In the early days of proteomics, 2D-GE-based studies were limited to the analysis of a few hundred proteins [140]. However, with the advent of MS-based approaches, the number of identified and quantified proteins has steadily risen. Modern high-resolution MS instruments, coupled with improved sample preparation and data analysis methods, can now routinely identify and quantify thousands of proteins in a single experiment [141].

In contrast, untargeted proteomic assays aim to capture the global protein composition of a sample, without any prior knowledge or selection of specific proteins [142]. These unbiased approaches, often referred to as “shotgun” or “discovery-based” proteomics, typically involve the use of data-dependent acquisition (DDA) or data-independent acquisition (DIA) MS techniques [143]. DDA methods rely on the automated selection of precursor ions for fragmentation, while DIA approaches systematically fragment all precursor ions within a defined mass range, allowing for the identification and quantification of a broader range of proteins [144]. Untargeted proteomic assays are well-suited for the discovery of novel biomarkers, the elucidation of disease mechanisms, and the exploration of complex biological systems [145].

#### 2.6.2. Multiplexed Immunoassays (Lumina Platforms)

In the 1990s, Luminex technology was first introduced and has since been widely adopted for targeted proteomic studies [146]. This technology uses color-coded magnetic or polystyrene beads coated with specific antibodies targeting different proteins. The beads are then incubated with the sample, allowing the proteins of interest to bind to their respective antibodies [147]. The Luminex assay offers several advantages over traditional ELISA methods, such as reduced sample volume, higher throughput, and the ability to measure multiple proteins in a single sample, and can typically measure up to 500 proteins simultaneously in a single sample [148]. The Luminex targeted proteomic approach has been widely used in various fields, including immunology, oncology, and infectious disease research [149]. These methods offer high sensitivity, specificity, and reproducibility, making them well-suited for known biomarker measurement or the validation of discovery-based findings [150].

#### 2.6.3. Integrating Proteomics and Beyond

The exponential growth in proteomic data has required sophisticated computational and bioinformatic tool development aiming for data processing, analysis, and interpretation. These include algorithms for peptide and protein identification, quantification, and statistical analysis, as well as tools for the integration of proteomic data with other “omics” datasets, such as genomics and metabolomics [151].

The process of discovering and developing new drugs has been revolutionized with the advent of omics sciences. Techniques such as cloning for recombinant protein expression and monoclonal antibodies, which combine genomics and proteomics, have enabled the precise and effective treatment of various diseases that have challenged doctors and researchers for decades [10]. Studies of monoclonal antibody therapy have led to a series of immunotherapeutic treatments for cancer. One of the major milestones was the use of monoclonal antibodies against human epidermal growth factor receptor 2 (HER2), which led to trastuzumab (Herceptin^®^; Roche Holding AG, Basel, Switzerland), the first product of its kind to be used successfully and safely against solid tumors [152,153]. The strategy of combining monoclonal antibodies and drugs has led to treatments for lymphoma and metastatic breast cancer [152]. A series of drugs based on monoclonal antibodies has also been used against autoimmune diseases such as rheumatoid arthritis, Crohn’s disease, multiple sclerosis, and psoriasis, among many others [154]. Infectious diseases that are difficult to treat using traditional methods are also prevented and treated with monoclonal antibodies, such as multidrug-resistant HIV infection, anthrax, and colitis caused by *Clostridium* [154].

The growth of the biological model field and the application of machine learning have also improved the capacity of understanding and even predicting protein structures using large public protein databases. The surge of AlphaFold 3 [155] brought high accuracy for protein–ligand, protein–nucleic acid and antibody–antigen interactions based on deep learning trained with the Protein Data Bank. Many other multi-chain deep-learning predictors were developed in the last two decades, culminating in the brand new D-I-TASSER [156].

Genomic and proteomic knowledge has laid the foundations for manipulating the genetic code and using non-coded and unnatural amino acids in peptide synthesis. As a result, engineered proteins with important experimental applications have already been obtained, such as copper-sensitive GFP fluorescent protein, human growth hormone (hGH) with increased serum half-life, antibodies with increased affinity, and enzymes with greater catalytic activity. In addition, non-coded and unnatural amino acids have led to major improvements in the production of biomaterials: the production of collagen-like protein with greater stability and promotion of angiogenesis; elastin-like protein with greater thermal stability and better mechanical properties; and silk-like protein with greater adhesion capacity. These conditions increase the possible applications of these materials in prosthesis development and tissue engineering [157].

### 2.7. Metabolomics

The concept of metabolic profiling, or metabolomics, was introduced in the late 1940s with the use of paper chromatography to suggest characteristic metabolic patterns in urine and saliva associated with diseases such as schizophrenia [158]. In the 1960s and 1970s, technological advancements in gas chromatography (GC), liquid chromatography (LC), and mass spectrometry (MS) allowed for quantitative metabolic profiling studies. In 1966, thin-layer chromatography (TLC) coupled with MS was used to analyze amino acids and other metabolites in urine samples [159]. Horning and co-workers successfully used GC-MS to measure metabolites in human urine and tissue [160]. This marked the beginning of the development of GC-MS-based techniques for metabolic measurements in biological fluids. Advancements in LC and GC-MS instrumentation and methods enable more sensitive, selective, and high-throughput metabolic profiling of biological samples [161].

The 1980s and 1990s saw significant advancements in MS instrumentation, including the introduction of high-resolution and high-sensitivity instruments, such as Nuclear magnetic resonance (NMR) spectroscopy [162]. This enabled the analysis of a wider range of metabolites and the development of more sophisticated analytical methods. The introduction of liquid chromatography-mass spectrometry (LC-MS) in the 1990s further expanded the capabilities of metabolomics [163].

In the 21st century, metabolomics experienced a significant improvement in popularity, mostly driven by advances in instrumentation and software [164,165]. Moreover, the development of databases such as METLIN and HMDB (Human Metabolome Database) [166,167], which contain MS/MS experimental data on over 930,000 molecular standards, has greatly facilitated metabolomic research. Other databases could be used, such as mzCloud (https://www.mzcloud.org/, accessed on 12 February 2026), containing high-resolution, accurate mass spectra of over 19,000 compounds, including 3700+ endogenous substances, or MassBank [168], a database primarily containing mass spectra obtained from chemical standards of metabolites. In addition to these databases, metabolite annotation can be further enhanced using classification databases like KEGG and others [169].

Since its inception, the metabolome has been closely related to other omics due to its dependence on data and analysis performed for these other sciences, especially transcriptomics, proteomics and pharmacogenomics. Gene expression analysis is a strategy to assess the biosynthesis of many metabolites by quantifying mRNAs associated with a metabolic pathway or specific molecule [170,171]. Thus, integrating this data with other metabolomic assessments is a useful way to identify genetic mechanisms that affect the metabolome of an individual [11].

The relation of metabolome to proteomics goes further, in the manner that its assays can also be classified into two categories: targeted and untargeted approaches. Targeted metabolomic assays involve the analysis of specific metabolites or groups of metabolites. These assays are typically used to study specific interventions’ effects or identify biomarkers for specific diseases [172]. Targeted assays are often more sensitive and specific than untargeted assays but may not provide a comprehensive view of the metabolome. Untargeted metabolomic assays involve the analysis of all metabolites present in a biological sample. These assays are typically used to study the global metabolic response to specific interventions or identify novel disease biomarkers. Untargeted assays are often less sensitive and specific than targeted assays but provide a more comprehensive view of the metabolome [173]. Taken together, both approaches could be used in the investigation of health–disease balance, drug efficacy and toxicity, and dietary effects [174].

### 2.8. Interactomics

The need for a deeper understanding of cellular functions, biological processes, coordinated responses to stimuli, and their consequences for the organism gave rise to interactomics, which investigates the complex and dynamic networks of interactions between proteins. Considered the most advanced stage of omics studies, the interactome consists of the set of interactions between proteins and other molecules, such as DNA, RNA, lipids, and other proteins at the cellular level [175,176]. From a clinical point of view, the definition of an interactome provides a powerful perspective for quantitatively and topologically identifying and classifying the biological processes involved in disease progression [177].

The first competent method for investigating the interactome was the yeast two-hybrid (Y2H) method. This technique attaches a bait to a transcriptional DNA-binding domain while the “prey” is attached to the transcriptional activation domain of the same transcriptional factor, and then both hybrid proteins are inserted into the same yeast strain. The result of the physical interaction between these two hybrid proteins is the expression of a reporter gene responsible for the yeast’s survival on media lacking a certain amino acid [178]. Although efficient in its purpose, Y2H is a very time-consuming and labor-intensive technique due to the need to select colonies from the media and use Sanger sequencing to identify prey fragments [179]. Recent updates on this method take advantage of modern deep-sequencing platforms to identify a number of interactors from Y2H experiments and generate interactomes on a genomic scale. This new approach was nominated yeast two-hybrid next-generation interaction screening (Y2H-NGIS) [180]. Both Y2H and Y2H-NGIS limit researchers, since they only allow the use of proteins that are not toxic to yeast [181].

An important advance in interactomics studies was the application of affinity purification coupled with the mass spectrometry technique (AP-MS). This method isolates protein complexes using a bait protein expressed with an epitope tag that will bind to other proteins in a complex, which are then purified by affinity. Those proteins are then identified using mass spectrometry [182]. AP-MS is being increasingly used to identify protein complexes due to the better efficiency in quantification, easier execution and more detailed results comprising dynamics, topology and stoichiometry [177,183]. Other advantages of the technique are the possibility of performing it in a way that maintains the physiological characteristics of the condition being studied, the conservation of post-translational modifications, and the visualization of dynamics in the composition of protein complexes [182]. A similar method applied for the identification of a proximity-based interactome is proximity-dependent biotin identification (BioID). Fusing the protein of interest with the mutated biotin ligase BirA* allows the covalent tagging of proteins in the surroundings with biotin, which is used for affinity purification and identification by mass spectrometry. The enzyme BirA* has an enhanced activity to catalyze the binding of biotin to proteins that interact directly or indirectly with the protein of interest, but also to other proteins located in the same subcellular region, also identifying weak and transient interactions [184].

Co-elution methods have been applied to interactomics studies and offer an improvement in many aspects over older methods. It provides information on the whole interactome of an individual instead of interactions of one protein at a time. Since proteins in the same complex migrate together in a purification column, this principle is applied to determine networks of all interacting proteins in a sample, using a separating method such as chromatography or blue-native polyacrylamide gel electrophoresis (BN-PAGE) [177].

### 2.9. Lipidomics

Lipidomics emerged as a new branch of metabolomics and has become the subject of debate regarding whether it is a new omics science. While also using targeted and untargeted approaches and relying largely on mass spectrometry to analyze a lipidic subset of molecules, some argue that the advent of lipidomics in the early 2000s provided the development of more specific strategies for studying these highly diverse macromolecules, leading to more in-depth findings and expanding the possibilities in clinical research [185,186,187]. Because hydrophobic molecules require analytical methods tailored to this characteristic, lipidomics has come to be recognized as the field responsible for analyzing the entire lipid profile, while metabolomics focuses broadly on hydrophilic metabolites [188].

The main methodological difference between lipidomics and metabolomics is the need for specific extraction methods tailored to the source material, due to the need to exclude polar molecules: butanol-methanol (BUME) uses a preliminary deproteinization step to isolate lipids from tissue and plasma samples [189]; methyl-tert-butyl ether (MTBE) is a less toxic alternative for cells in this same type of sample [190]; in turn, the Folch method is the oldest and still the most widely used for all types of samples [191]; the Bligh–Dyer method, on the other hand, modifies the Folch method with a focus on cell culture samples [192].

The analytical phase of lipidomics essentially shares the strategies and platforms of metabolomics. The approach can be targeted, focusing on the quantification of specific, well-defined lipids, or untargeted, capable of screening a broader range of known and unknown lipids. The use of chromatography, ionization, and mass spectrometry occurs as in metabolomics, with an emphasis on the preferential application of MALDI in solid samples [188]. These two decades of development and application in the field have already led to significant advances in clinical studies aimed at better understanding and monitoring metabolic disorders, cardiovascular and neurological diseases, and cancers such as breast and prostate cancer [187,193].

### 2.10. Glycomics

Another subset of macromolecules, glycans, are the focus of glycomics. This omic science dwells on the composition, structure, and functions of these important molecules [194]. Once considered the “dark matter of biology”, glycans are progressively gaining attention for their role in pathogen–host interactions and as a biomarker for multiple diseases, like cancer and kidney diseases [195,196,197,198].

Like metabolomics and lipidomics, glycomics predominantly uses mass spectrometry as a highly sensitive analytical method. Due to the molecular characteristics of glycans, additional techniques are required for the identification of isomers, both before and after mass spectrometry. Ionization methods such as MALDI-(TOF)MS combined with chemical derivatization, or dissociation methods such as ultraviolet photodissociation (UVPD), electron-transfer dissociation (ETD), collision-induced dissociation (CID), and higher-energy collisional dissociation (HCD) are commonly used for their effective application in characterizing glycans and their isomers [199,200]. Since it is impossible to fully understand the functions of glycans in isolation, it is often necessary to integrate glycomics and proteomics in order to analyze glycoprotein patterns, an approach known as glycoproteomics [201].

## 3. Lord of the Omics: The Society of Personalized Medicine—Clinical Implementation of Precision and Personalized Medicine

The broad and integrative nature of the omics sciences has led to the application of their principles to practical approaches in medicine, exemplified essentially by precision medicine and personalized medicine. Precision medicine integrates different types of clinical data from patients in order to identify the best choice of treatment for a patient based on their genetic and phenotypic characteristics. The last decade has shown substantial advances in this area, contributing to the consolidation of knowledge in diseases of different natures. Studies have laid the foundations for new ways of diagnosing and treating different types of cancer [202,203], type 1 and type 2 diabetes [204,205], sepsis [206], diseases of the central and peripheral nervous system [207,208,209] and cystic fibrosis [210].

For its part, personalized medicine combines genetic information with the monitoring of a large amount of clinical information on each patient to gain a deeper insight into the individual’s health. The proposed interventions involve advanced techniques such as the application of AI to clinical tests for diagnosis, bioengineering and nanotechnology, genetic engineering and gene editing for the treatment of complex diseases like tuberculosis, HIV and cancer [211]. Although still in their infancy, examples of the application of personalized medicine to solve fertility problems in men and women have shown promising results [212,213].

## 4. Lord of the Omics: The Return of Machine Learning—Persistent Bottlenecks, Computational Horizons, and Ethical Challenges

Many technical and technological advances made in recent years can push the omics science forward in terms of timing, precision and productivity. The application of cutting-edge deep-learning algorithms in the analysis of large biological datasets is already helping health professionals in diagnosis and preventive medicine. Researchers have developed a predictive machine learning model based on random forest to aid in the diagnosis of malaria [214]. The differential diagnosis of causes of vertigo, a historically complex topic, has also been the target of approaches that apply machine learning. Thus, they allow information such as background, pre-existing symptoms, exams, and laboratory tests of individuals to support more accurate decisions by healthcare professionals [215]. However, clinical cancer research stands out in the robustness and diversity of studies using artificial intelligence, applying a series of machine learning methods for risk stratification and prediction, diagnosis, prognosis, and treatment strategy selection [216].

While they are already being used in cutting-edge hospitals, precision medicine and personalized medicine still have a long way to go in terms of applications and availability with the evolution of the omics sciences. Although promising, personalized medicine and precision medicine still face crucial challenges. The speed of technological advances is not yet sufficient to make these approaches available to the general public due to their current high cost, which still makes them considerably disadvantageous to public and private health systems compared to traditional approaches. Considering that precision medicine primarily requires a large amount of genetic data, medical reports and clinical information at a population level to profile and identify patterns of disease development in order to develop individualized treatments, the constant progress of biological sample processing platforms and the increase in computing power available to researchers vow to promote a rapid advance in the efficiency and popularization of this approach [217].

Despite the evolution of techniques for obtaining and analyzing data from various omics, which contribute enormously to scientific knowledge and to solving clinical situations, it also brings new persistent challenges, especially for bioinformaticians. The generation of biological data, facilitated by the lowering of technology cost, is becoming accessible to more and more research groups around the world [53]. As a result, a massive amount of data fills public databases, even though it lacks considerable metadata, quality control and precise information about the experiment that generated it. Failure to meet these requirements compromises the reproducibility of analyses and the use of data for other purposes [218]. This reuse of publicly available data is both a challenge and an opportunity for bioinformaticians. In view of the generation of a large amount of biological data aimed at answering a single scientific question, this allows for a multitude of subsequent approaches by various other research groups, which can contribute to expanding knowledge in the omics sciences without the need to generate new data.

Although the Human Genome Project is a landmark not only for the omics sciences, but also for health professionals and clinical researchers in general, it is still arguable how much of its impact has reached the general population yet, and how long it will take until such advances made since then will become available in healthcare systems around the world, especially for ethnic minorities and developing countries [219]. Combining this with the fact that 91% of genome-wide association studies (GWAS) have been conducted on individuals of European ancestry, while less than 4% of available samples come from developing countries, we see an imbalance that compromises equity in the development of health practices and access to modern and accurate treatments [220].

These issues must be considered before and during the process of designing scientific studies, developing new equipment and technologies, and constructing policies for implementing new public health approaches based on omics sciences. As for clinical studies, it is essential to increase the ethnic and geographic representativeness of the data analyzed, taking into account the impact of genetic variability and cultural diversity among different populations on epidemiology, acceptance of medical interventions, and the effectiveness of clinical treatments. Technological progress must be measured in terms of economic accessibility, allowing for the democratization of the most modern and effective methods. In turn, public and private health policies must focus on making practices based on omics science knowledge available to broad populations, including in developing countries.

Ethical issues related to access, use, and storage of patient data and the use of gene editing techniques and stem cells also remain legal obstacles in many countries. The sensitive nature of fundamental issues for these technologies, such as the concept of embryonic life and human genome modification, means that the ethical precepts surrounding their use, application, and even clinical studies are permeated by cultural and religious elements. As a result, it becomes complex to develop common and concise legal guidelines, creating disparities between countries and delaying scientific progress in the field [221]. The record of ethical deviations in the use of genetic modification techniques also contributes to reinforcing conservative positions on the use of these technologies and highlights the need for clear and well-defined rules to regulate healthy scientific development [222]. The generation of genomic data from individuals, from personalized medicine to ancestry testing, also raises the need for strong legal regulation to maintain the privacy of such sensitive information, which is coveted by health insurers for commercial purposes [222].

Future prospects for the application of omics sciences in clinical studies and health sciences suggest the gradual replacement of the traditional model of medicine, which applies standardized treatments to all patients with a given condition, with personalized strategies tailored to the particularities of each individual, based on increasingly vast and detailed omics data. To achieve this, it will be essential to overcome current challenges, such as the accuracy of the data generated, patient access to the necessary infrastructure, and mitigation of human and computational bottlenecks in data analysis [213].

## Figures and Tables

**Table 1:** Historical overview of DNA sequencing technologies and their impact on omics science.

Sequencing Generation	Main Principle	Key Characteristics	Representative Platforms/Methods	Major Applications
First-generation sequencing (1960s–1970s)	Chemical cleavage or chain termination during DNA synthesis	Low throughput; high cost; labor-intensive; initially relied on radioactive labeling; high accuracy	Chemical sequencing; Sanger sequencing	Early gene sequencing; method development; foundation of molecular genetics
Second- generation sequencing—NGS (2005-)	Massively parallel sequencing of short DNA fragments	High throughput; short read lengths (100–600 bp); reduced cost per base; requires DNA amplification and fragmentation	Illumina HiSeq; Illumina MiSeq	Whole-genome sequencing; metagenomics; microbiome studies; disease gene panels; pharmacogenomics; personalized medicine
Third- generation sequencing—TGS (2010-)	Real-time sequencing of single DNA molecules	Long read lengths (thousands of bp); no amplification required; reduced bias; detection of structural variants and epigenetic modifications	PacBio Sequel; Oxford Nanopore MinION	De novo genome assembly; structural variation analysis; epigenomics; clinical and field-based genomics

**Table 2:** Classification of biomarkers and their roles in precision medicine.

Classification	Category	Definition	Examples	Main Applications
Biological characteristics	Molecular	Biomarkers derived from DNA, RNA, proteins, or metabolites	BRCA1/2 mutations; circulating miRNAs; plasma metabolites	Disease risk assessment; diagnosis; therapy selection
	Histological	Tissue-based structural or cellular features	Tumor grade; Gleason score	Cancer diagnosis and prognosis
	Radiographic	Imaging-based biomarkers	MRI lesions; CT scan findings; X-ray abnormalities	Disease detection; monitoring progression
	Physiological	Functional measurements of biological systems	Blood pressure; heart rate; glomerular filtration rate	Disease monitoring; treatment evaluation
Information provided	Exposure biomarkers	Indicate exposure to an external agent and its extent	Cotinine levels (nicotine); blood lead levels	Environmental and occupational health assessment
	Effect biomarkers	Reflect biological changes associated with disease or intervention	Elevated liver enzymes; inflammatory markers	Early disease detection; treatment monitoring
	Susceptibility biomarkers	Indicate predisposition to disease due to genetic or biological factors	BRCA1/2 mutations; inherited risk alleles	Risk stratification; preventive strategies
Clinical role	Diagnostic biomarkers	Detect or confirm the presence of a disease or define disease subtypes	HbA1c (diabetes); BRCA1/2 (breast cancer); GFR (CKD)	Disease diagnosis; patient stratification
	Prognostic biomarkers	Predict disease outcome regardless of treatment	BRAF V600E (melanoma); Gleason score (prostate cancer)	Outcome prediction; clinical decision-making
	Predictive biomarkers	Predict response to a specific therapy	Estrogen receptor (breast cancer); CD20 (lymphomas)	Personalized treatment selection
	Monitoring biomarkers	Track disease status or treatment response over time	HIV viral load; body weight changes	Therapy adjustment; disease control
	Pharmacodynamic biomarkers	Indicate biological response to a therapeutic intervention	Glycated hemoglobin during antidiabetic therapy	Drug efficacy assessment

## Data Availability

No new data were created or analyzed in this study. Data sharing is not applicable to this article.
